# *Vibrio campbellii hmgA*-mediated pyomelanization impairs quorum sensing, virulence, and cellular fitness

**DOI:** 10.3389/fmicb.2013.00379

**Published:** 2013-12-11

**Authors:** Zheng Wang, Baochuan Lin, Anahita Mostaghim, Robert A. Rubin, Evan R. Glaser, Pimonsri Mittraparp-arthorn, Janelle R. Thompson, Varaporn Vuddhakul, Gary J. Vora

**Affiliations:** ^1^Center for Bio/Molecular Science & Engineering, Naval Research LaboratoryWashington, DC, USA; ^2^School of Systems Biology, College of Science, George Mason UniversityFairfax, VA, USA; ^3^Mathematics Department, Whittier CollegeWhittier, CA, USA; ^4^Division of Electronics Science and Technology, Naval Research LaboratoryWashington, DC, USA; ^5^Department of Microbiology, Faculty of Science, Prince of Songkla UniversityHat Yai, Thailand; ^6^Department of Civil and Environmental Engineering, Massachusetts Institute of TechnologyCambridge, MA, USA

**Keywords:** *Vibrio*, melanin, bioluminescence, quorum sensing, tyrosine catabolism

## Abstract

Melanization due to the inactivation of the homogentisate-1,2-dioxygenase gene (*hmgA)* has been demonstrated to increase stress resistance, persistence, and virulence in some bacterial species but such pigmented mutants have not been observed in pathogenic members of the *Vibrio* Harveyi clade. In this study, we used *Vibrio campbellii* ATCC BAA-1116 as model organism to understand how melanization affected cellular phenotype, metabolism, and virulence. An in-frame deletion of the *hmgA* gene resulted in the overproduction of a pigment in cell culture supernatants and cellular membranes that was identified as pyomelanin. Unlike previous demonstrations in *Vibrio cholerae, Burkholderia cepacia*, and *Pseudomonas aeruginosa*, the pigmented *V. campbellii* mutant did not show increased UV resistance and was found to be ~2.7 times less virulent than the wild type strain in *Penaeus monodon* shrimp virulence assays. However, the extracted pyomelanin pigment did confer a higher resistance to oxidative stress when incubated with wild type cells. Microarray-based transcriptomic analyses revealed that the *hmgA* gene deletion and subsequent pyomelanin production negatively effected the expression of 129 genes primarily involved in energy production, amino acid, and lipid metabolism, and protein translation and turnover. This transcriptional response was mediated in part by an impairment of the quorum sensing regulon as transcripts of the quorum sensing high cell density master regulator LuxR and other operonic members of this regulon were significantly less abundant in the *hmgA* mutant. Taken together, the results suggest that the pyomelanization of *V. campbellii* sufficiently impairs the metabolic activities of this organism and renders it less fit and virulent than its isogenic wild type strain.

## Introduction

As a member of the L-tyrosine catabolism pathway in bacterial and eukaryotic organisms, the enzyme homogentisate 1,2-dioxygenase (HmgA) catalyzes the intermediate homogentisic acid into 4-maleylacetoacetate which is further catabolized to yield fumarate and acetoacetate. In some bacterial species, it has been demonstrated that the inactivation of the *hmgA* gene results in the accumulation of homogentisic acid which when auto-oxidized leads to the formation of the water-soluble brown pigment pyomelanin (Rodriguez-Rojas et al., 2009; Schmaler-Ripcke et al., 2009; Turick et al., 2009; Valeru et al., 2009; Wang et al., 2011). This phenotype has been observed in naturally pigmented environmental and clinical strains of *Vibrio cholerae* and has been shown to be due to mutations in the *hmgA* gene (Wang et al., 2011). Interestingly, pyomelanin pigmented *V. cholerae* demonstrate greater UV and oxidative stress resistance, virulence factor expression and infant mouse intestine colonization rates than their non-pigmented counterparts (Valeru et al., 2009). The ability of pyomelanin to confer increased resistance to oxidative stress appears to contribute to virulence by reducing the susceptibility of pigmented bacteria to host defense mechanisms. Because of these particular characteristics, it is not surprising that pyomelanin-producing *Pseudomonas aeruginosa* and *Burkholderia cepacia* are frequently isolated from cystic fibrosis patients (Zughaier et al., 1999; Rodriguez-Rojas et al., 2009). Furthermore, the production of pyomelanin has also been shown to provide greater protection from other environmental stresses such as hyperosmotic shock and elevated temperatures (Kotob et al., 1995) and act as a sole terminal electron acceptor and soluble electron shuttle to iron which may provide an additional fitness advantage to pyomelanin-producing mutants in anaerobic environments (Turick et al., 2002).

Despite these seemingly advantageous phenotypes, such pigmented mutants have not been reported from pathogenic members of the *Vibrio* Harveyi clade. Two of the most economically important Harveyi clade species, *V. campbellii* and *V. harveyi*, are common inhabitants of tropical marine environments and are among the most important bacterial pathogens of many commercially farmed marine invertebrate and vertebrate species (Thompson et al., 2004; Austin and Zhang, 2006). As certain pathogenic members of both species are capable of producing quorum sensing induced bioluminescence, the disease caused by them is often referred to as luminescent vibriosis (Defoirdt et al., 2008) and is a disease manifestation that is frequently implicated in outbreaks within penaeid shrimp larval culture facilities worldwide (Austin and Zhang, 2006). Given the importance of shrimp hemocyte-mediated oxidative defense mechanisms in combatting *Vibrio* infections (Ji et al., 2011), it is not unreasonable to posit that pyomelanization may benefit the survival and perhaps exacerbate the virulence of vibrios in this host environment. However, the production of pyomelanin comes at the cost of impairing the tyrosine catabolism pathway and the effect of the inactivation of *hmgA* and/or pyomelanin production on global cellular metabolism is not known. In this study, we used *V. campbellii* ATCC BAA-1116, a bioluminescent marine bacterium that is best known as a model organism for quorum sensing studies (Bassler, 1999), to begin to determine the generality of pyomelanin-mediated phenotypes and how the deletion of the *hmgA* gene and resulting pyomelanin production may affect cellular phenotypes, virulence, and transcription.

## Materials and methods

### Bacterial strains and growth conditions

*V. campbellii* (ATCC strain BAA-1116; previously known as *V. harveyi* BAA-1116 or BB120; Lin et al., 2010) and the *hmgA* mutant were grown in Luria Marine (LM) medium (20 g NaCl, 10 tryptone, 5 g yeast extract per L, pH 7.8) or Tryptic Soy Broth containing 1% NaCl. *Escherichia coli* DH5α and SM10λ pir used for standard DNA manipulation and conjugation were grown in Luria Broth (LB) medium.

### Construction of the hmgA in-frame deletion mutant

The in-frame deletion of the *V. campbellii hmgA* gene (Δ*hmgA*) was generated by overlap PCR (Warrens et al., 1997). Briefly, ~500 bp DNA fragments upstream and downstream of the *hmgA* open reading frame were amplified from *V. campbellii* BAA-1116 genomic DNA using the primer pairs *hmgA*-a (5′-TAggatccTGTACGAAATCGACCATCTGAC)/*hmgA*-b (5′-c) and *hmgA*-c (5′-GAGGAGTACTAAGCGGGGGCAAGGATGAAA)/*hmgA*-d (5′-CActcgagACTTCACCTTCGAAGTCAATCC), respectively. The two PCR products were annealed using their overlapping region and amplified using primers *hmgA*-a and *hmgA*-d. The resulting 1 Kb PCR fragment was cloned into the pCR4-TOPO vector using the TOPO TA cloning kit (Invitrogen, Carlsbad, CA, USA). This assembled fragment was then digested from the TOPO vector with *Bam*HI and *Xho*I and cloned into the plasmid pZW125, which was constructed by inserting a chloramphenicol resistance gene into the oriR_*R*6*Kg*_ plasmid pWM91 containing the *sacB* gene (Metcalf et al., 1996). The resulting plasmid (pZW025) was transformed into *E. coli* strain Sm10λ pir and transferred into a *V. campbellii* spontaneous streptomycin resistant mutant (*V. campbellii*-str1) by conjugation. The conjugants were grown on LM agar plates containing 3 μg/mL chloramphenicol and 1 μg/mL streptomycin. The Δ*hmgA* was selected on LM plates supplemented with 6% sucrose and verified by PCR.

### Growth curve analyses

Bacterial replication was measured using a Bioscreen C analyzer (Growth Curves USA, Piscataway, NJ, USA). Briefly, overnight cultures were diluted 1:5000 (~10^5^ cells/mL) in pre-warmed LM and five 200 μL aliquots of the wild type (WT) and Δ*hmgA* strains were transferred into a 100-well honeycomb plate. The plate was incubated at 30°C for 48 h with continuous shaking and wide band OD_450−580 *nm*_ measurements taken every 30 min. Three independent experiments were performed in this manner.

### Measurement of pigmentation, bioluminescence and cellular susceptibility to H_2_O_2_

Matched diluted overnight WT and Δ*hmgA* cultures were used to inoculate 50 mL LM media in 250 mL flasks and incubated at 30°C and 200 rpm. Every 24 h, three 100 μL aliquots of culture were collected and bacterial cells were pelleted via centrifugation at 10,000× *g* for 5 min. Supernatant pigments were measured using a NanoDrop ND-2000c spectrophotometer (Thermo Scientific, Pittsburg, PA, USA) at OD_400_. Another three 100 μL aliquots of culture were placed in a black U96 Nunc MicroWell™ plate (Thermo Scientific) and measured for bioluminescence using a Luminoskan Ascent Microplate Luminometer (Thermo Scientific). Three independent experiments were performed in this manner.

At the 48 h time point, WT, and Δ*hmgA* cells were harvested, washed and resuspended in fresh LM media. They were then incubated with 2 mM H_2_O_2_ at room temperature for 15 min. The percentage survival was calculated by counting colony forming units (CFU) immediately before and after the H_2_O_2_ treatment on LM agar plates. The potentially protective effect of WT and Δ*hmgA* culture supernatants against H_2_O_2_ treatment was also tested using WT cells. Briefly, mid-log phase WT cells were harvested, washed and resuspended in 0.2 μm filter-sterilized supernatants from WT and Δ*hmgA* 48 h LM media cultures*.* The cell suspensions were then incubated at room temperature in the presence of 2 mM H_2_O_2_ for 15 min. The percentage survival was calculated by counting CFUs immediately before and after the H_2_O_2_ treatment on LM agar plates. The data for each of these experiments was generated from three independent assays.

### Pigment preparation and electron spin resonance spectroscopy

Partial purification of the pigment from the Δ*hmgA* strain was modified from the method previously described by Turick et al. (2002). Briefly, a 50 mL Δ*hmgA* culture was grown in LM at 30°C for 96 h with shaking at 200 rpm. The cells were harvested via centrifugation at 5000× *g* for 10 min, and the supernatant was removed and acidified with 6 N HCl to a final solution concentration of 0.4 N and was then allowed to precipitate for 12 h at room temperature. The concentrated pigment was collected by centrifugation at 8000× *g* for 20 min, washed twice with dH_2_O and then dried using a SpeedVac Concentrator (Thermo Scientific). A pure synthetic melanin that was chemically prepared from the oxidation of tyrosine was purchased and used as a control (M8631, Sigma-Aldrich, St. Louis, MO, USA). A second control, DHN-melanin, was prepared from the conidia of the fungus *Aspergillus niger* using the method of Youngchim et al. (2004). The pigment powder samples were characterized by electron spin resonance (ESR) at 300K in a Bruker 9.5 GHz spectrometer. Typical microwave powers of 5–20 mW with 1G modulation amplitude and 100 kHz field modulation were employed for these experiments.

### Shrimp virulence assays

The LD_50_ of the WT and Δ*hmgA* strains were evaluated on the black tiger shrimp *Penaeus monodon*. Both strains were grown in Tryptic Soy Broth containing 1% NaCl at 30°C with shaking at 150 rpm, harvested by centrifugation at 2000× *g* for 10 min and washed twice with sterile Marinum® artificial seawater (ASW) (Mariscience International Co. Ltd., Bangkok, Thailand). Bacterial cell suspensions in ASW were adjusted to 2.6 × 10^8^ CFU/mL using a turbidimeter (Oxoid Ltd., United Kingdom) and twofold dilutions were performed to obtain the required concentrations of bacteria prior to injecting the shrimp. The juvenile shrimp used in this study were 10–13 g in weight and 4–5 inches in length. Each shrimp received an intramuscular injection of 100 μL diluted *V. campbellii* (with batches of seven shrimp/dose) between the third and fourth abdominal segments. Control shrimp were injected with ASW. The experiments were performed in quadruplicate. The animals were maintained in a 70 L ASW glass tank at a temperature of 29 ± 1°C and salinity of 17 ppt. Shrimp mortalities were observed within 48 h of injection and were confirmed by detecting bioluminescence in the organs of the dead shrimp. The LD_50_ was calculated using the method of Reed and Muench (1938).

### Microarray-based transcriptome analyses

Aliquots of three cultures (3.0 × 10^8^ cells/mL) of the WT and Δ*hmgA* strains grown in LM at 30°C for 48 h with constant shaking at 200 rpm were harvested for total RNA extraction. RNA was isolated using the RiboPure™-Bacteria Kit (Life Technologies, Grand Island, NY, USA), treated with DNase and 10 μg of total RNA from each culture was further purified using the MICROB*Express*™ Bacterial mRNA Enrichment Kit (Life Technologies) according to the manufacturer's specifications. All RNA preparations were quantified and analyzed using the Agilent 2100 Bioanalyzer (Agilent Technologies, Inc., Santa Clara, CA, USA) and normalized to 1 μg. The normalized RNA was labeled, purified, fragmented and hybridized to a custom Affymetrix microarray (520694F) according to standard protocols (Affymetrix, Santa Clara, CA, USA). All hybridizations incubated for 16 h at 49°C in the GeneChip® Hybridization Oven 640 at 60 rpm and the microarrays were then washed and stained with the GeneChip® Fluidics Station 450 and scanned using the GeneChip® Scanner 7G (Affymetrix). Hybridization signal intensities were analyzed with the GeneChip® Operating Software (GCOS) to generate raw image files (.DAT) and simplified image files (.CEL) with intensities assigned to each of the corresponding probe positions. The data collected was used to profile the expression levels of 4831 open reading frames. The data containing the distribution of the probe amplitudes were calculated and a classical analysis of variance (ANOVA; applying the CRAN R's aov function) was performed across conditions for each probe site. The median values revealing the gene-level measurement of differential expression was determined (Rubin, 2009). The gene designations and annotations utilized are from the Naval Research Laboratory's *V. campbellii* ATCC BAA-1116 genome sequencing effort (GenBank accession numbers CP006605, CP006606, CP006607) and the expression profiling data can be found in the GenBank Gene Expression Omnibus repository (accession number GSE46223).

### Quantitative reverse transcription PCR

Real-time reverse transcription PCR assays were conducted using the iScript™ One-Step RT-PCR Kit with SYBR Green (Bio-Rad Laboratories, Hercules, CA, USA). One nanogram of mRNA from two biological replicates were tested in triplicate on an iCycler (BioRad). The PCR primers were designed using Primer3 online software (v. 0.4.0) (http://frodo.wi.mit.edu/). Relative quantities of the transcripts were determined using the 2^−ΔΔ^Ct formula where ΔCt is the difference in Ct of the selected genes and Ct of the normalizer gene, and ΔΔCt is the difference in ΔCt from Δ*hmgA* and ΔCt from the WT. The *rpoS1* gene was used to normalize the expression levels of the selected genes as its transcription level was found to be constant in both the WT and Δ*hmgA* strains.

## Results and discussion

### Characterization of ΔhmgA pigment and phenotypes

**Figure 1:**
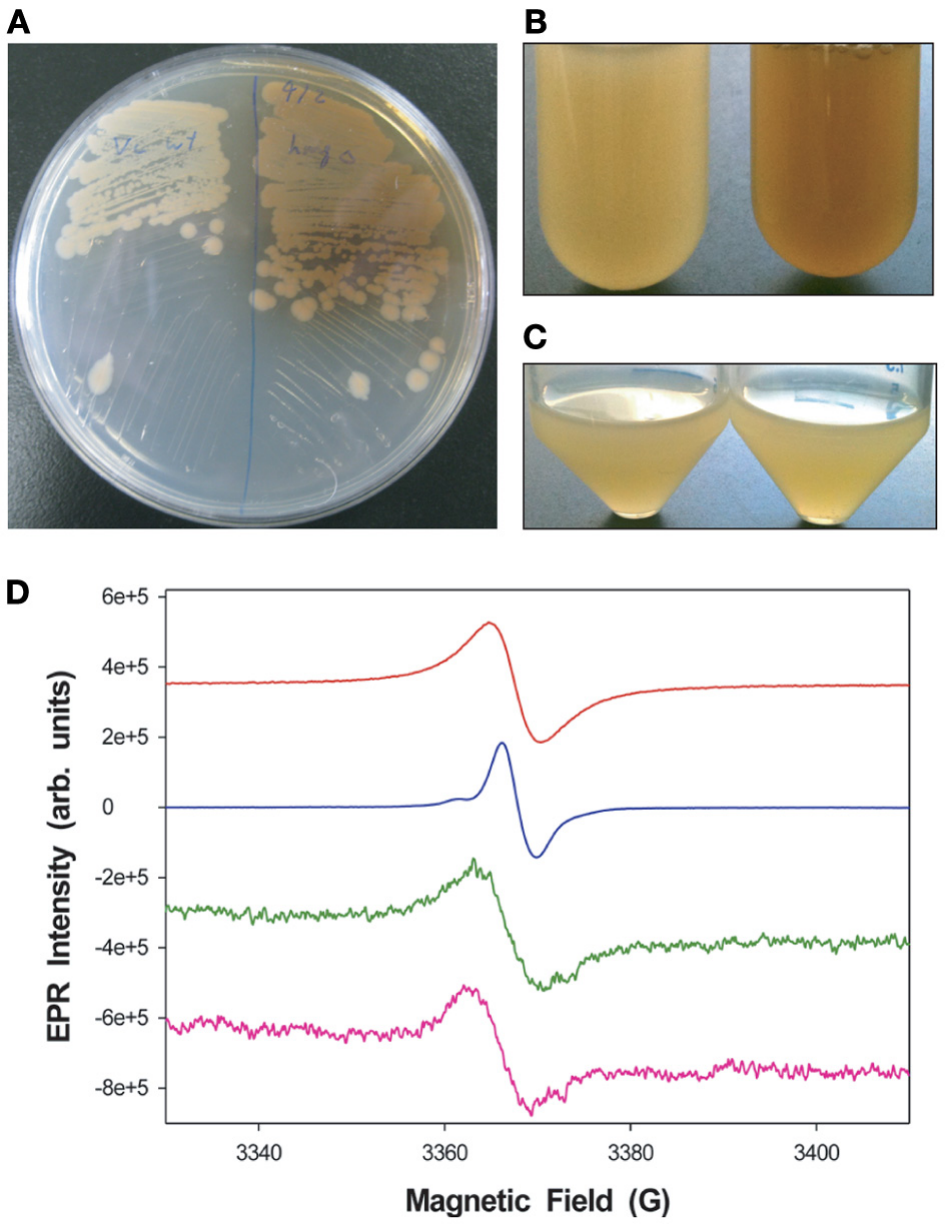
**Pigmentation differences between *V. campbellii* WT and Δ*hmgA*. (A)** Colonies grown on LM agar. WT (left), Δ*hmgA* (right); **(B)** Cell cultures grown in LM in borosilicate glass tubes (48 h). WT (left), Δ*hmgA* (right); **(C)** Cell cultures grown in LM in polypropylene conical tubes (48 h). WT (left), Δ*hmgA* (right); **(D)** EPR spectra of synthetic melanin (red), DHN-melanin (blue) and partially purified pigments from the supernatants of two independent Δ*hmgA* cultures (green and pink).

An examination of the *V. campbellii* ATCC BAA-1116 genome revealed that 4 genes in the catabolic pathway of tyrosine metabolism, *hmgA* (M892_02450), *hppd* (4-hydroxyphenylpyruvate dioxygenase, M892_02455), *fahA* (fumarylacetoacetase, M892_02445), and *maiA* (maleylacetoacetate isomerase, M892_02440), appeared to form an operon and shared the same genetic synteny as other sequenced *Vibrio* species. An in-frame deletion of the *hmgA* gene was generated (Δ*hmgA*) to investigate the role of this gene in pigment production and cellular physiology. When cultured on LM agar plates, in baffled glass Erlenmeyer flasks or polystyrene round-bottomed tubes for ≥ 48 h, the Δ*hmgA* strain produced a brown pigment that was expected to be the result of the auto-oxidation and polymerization of homogentisate (Figures 1A,B). The pigment was found to be present in Δ*hmgA* cell-free supernatants as well as washed cell pellets suggesting that the pigment was not only released into the microenvironment but could also be found associated with the bacterial cell membrane (data not shown). Interestingly, the production of this pigmentation was not observed when Δ*hmgA* cells were cultured in 50 mL polypropylene conical tubes (Figure 1C). Under the experimental conditions utilized, the 50 mL conical tubes provided the least aeration. In the absence of sufficient aeration, which is required for the oxidation of homogentisate, the production of the pigment was not observed. In order to identify the oxidized homogentisate polymer, the extracted brown pigment from Δ*hmgA* cell culture supernatants was examined using ESR. The ESR spectrum of the pigment revealed a distinct stable free radical signal that was characteristic of melanin and highly similar to synthetic eumelanin and DHN melanin of *Aspergillus niger* (Figure 1D) thus, confirming that the Δ*hmgA* pigment was pyomelanin. The results demonstrate that like *V. cholerae* Δ*hmgA*, *V. campbellii* Δ*hmgA* also produces pyomelanin but does not appear to do so in the same abundance or nearly as rapidly (Valeru et al., 2009).

**Figure 2:**
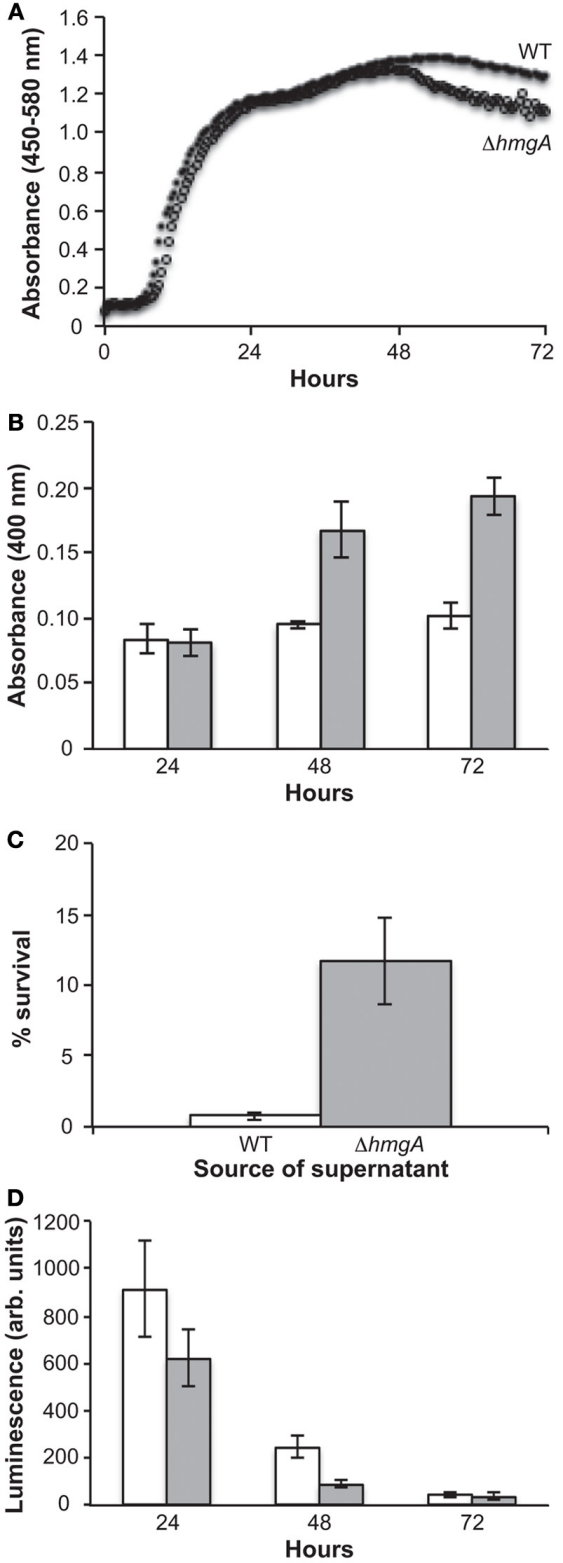
**Growth, pigment production and bioluminescence in *V. campbellii* WT and Δ*hmgA*. (A)** Grow curves of the WT and Δ*hmgA* strains in LM at 30°C. WT (closed circle), Δ*hmgA* (open circle); **(B)** Absorbance of pigment-containing supernatants. WT (white bars), Δ*hmgA* (gray bars); (**C**) Survival of WT *V. campbellii* cells in 2 mM H_2_O_2_ when resuspended in supernatants from WT or Δ*hmgA* cultures; **(D)** Bioluminescence measurements. WT (white bars), Δ*hmgA* (gray bars). Error bars represent the standard deviation from three experiments.

Growth curve analyses in nutrient rich LM medium revealed that the WT and Δ*hmgA* strains grew equally well during the lag, log, and early stationary phases of growth. However, Δ*hmgA* displayed a lesser ability to survive during late stationary phase (post 48 h) (Figure 2A): a time point that coincided with the measurable production of pyomelanin (Figure 2B).

In other bacterial and fungal organisms, melanin has been demonstrated to have a role in protecting against certain environmental stressors as mutations in the *hmgA* gene and the resulting melanization have been shown to provide an increased resistance to UV irradiation and H_2_O_2_-mediated oxidative stress (Rodriguez-Rojas et al., 2009; Schmaler-Ripcke et al., 2009; Valeru et al., 2009). However, when we performed similar experiments comparing the response of *V. campbellii* WT and Δ*hmgA* washed cells to different doses of UV irradiation and concentrations of H_2_O_2_, no significant differences in viability were observed (data not shown). Interestingly, however, we did observe the protective properties of pyomelanin when cell-free pyomelanin-containing supernatants from Δ*hmgA* cultures were incubated with WT cells and then exposed to 2 mM H_2_O_2_ (Figure 2C). Comparatively, cell-free supernatants from WT cultures did not demonstrate this protective function. The combined H_2_O_2_ challenge results demonstrated the protective material property of *V. campbellii* pyomelanin and suggest that there is not sufficient membrane-associated pyomelanin in *V. campbellii* to afford this same level of protection in the absence of the accumulated extracellular pyomelanin.

Unlike any other pyomelanin-producing bacterium investigated to date, *V. campbellii* ATCC BAA-1116 is capable of generating quorum sensing-induced bioluminescence. Interestingly, previous studies have suggested that the luciferase enzyme and bacterial bioluminescence, like pyomelanin, also play a physiological role in protecting cells against UV and oxidative stress. For example, UV irradiation has been found to stimulate bioluminescence (Czyz et al., 2002) which in turn photoreactivates DNA repair processes (Kozakiewicz et al., 2005) and bioluminescent cells have been shown to be significantly more resistant to UV irradiation than their non-bioluminescent counterparts (Czyz et al., 2000; Kozakiewicz et al., 2005). In addition, various oxidants, such as H_2_O_2_, have been shown to severely impair the growth of vibrios lacking the luciferase enzyme (Szpilewska et al., 2003). This is due to the fact that in addition to the production of light, this enzyme is also capable of increasing cellular resistance to oxidative stress by detoxifying reactive oxygen species (Katsev et al., 2004). Therefore, in addition to the more common bacterial mechanisms of UV and oxidative stress protection, bioluminescent *V. campbellii* also contain luciferase-based protective mechanisms. This combination of luciferase and pyomelanin in the same organism introduced the possibility of additive or synergistic stress protection. However, when the bioluminescence output of Δ*hmgA* was examined during measurable pyomelanin production (48 h), it was found to be significantly attenuated in comparison to the WT (Figure 2D). While this result demonstrated a diminution of light production in pyomelanin-producing Δ*hmgA*, it was incapable of determining whether this was due to a decrease in luciferase abundance or activity, reduced intracellular O_2_ or necessary substrates.

### Shrimp virulence model

In *V. cholerae* Δ*hmgA* and *P. aeruginosa* Δ*hmgA*, pyomelanization was shown to play a role in increasing virulence factor expression and adaptation to chronic infections in vertebrate animal models (Rodriguez-Rojas et al., 2009; Valeru et al., 2009). As *V. campbellii* BAA-1116 is known to pathogenize shrimp, we sought to determine whether *V. campbellii* pyomelanization would have a similar effect on invertebrate animal infections. *V. campbellii* WT and Δ*hmgA* were used to infect juvenile black tiger shrimp (*Penaeus monodon*) and the LD_50_ of both were evaluated. Surprisingly, the LD_50_ of Δ*hmgA* was ~2.7 times higher (less lethal) than that of the WT indicating that the production of pyomelanin in Δ*hmgA* was associated with decreased virulence in this model infection system (Table 1).

**Table 1:** ***V. campbellii* BAA-1116 and Δ*hmgA Penaeus monodon* challenge**.

**Experiment no**.	**LD_50_ (CFU/mL)**	
	**WT**	**Δ*hmgA***
1	1.4 × 10^8^	> 3.1 × 10^8^
2	2.1 × 10^8^	4.1 × 10^8^
3	2.4 × 10^8^	5.8 × 10^8^
4	1.9 × 10^8^	5.4 × 10^8^
Mean ± SD	1.9 ± 0.4 × 10^8^	5.1 ± 0.9 × 10^8^

Four independent LD_50_ experiments were performed. Mean values of LD_50_ between the WT and ΔhmgA were significantly different (P < 0.05, student's t-test).

In invertebrates, one of two major immune responses against invading pathogens is the prophenoloxidase activating system (proPO) (Cerenius and Soderhall, 2004). Upon infection, non-self molecules such as lipopolysaccharide, peptidoglycan and β-glucan can activate the proPO I cascade and result in the formation of melanin around the invading microorganisms. In this circumstance, the host formed melanin is thought to physically shield the pathogens to prevent or retard their growth. Host derived quinones, which are intermediates of melanin production, may also be involved in the production of cytotoxic molecules (e.g., superoxides, hydroxyl radicals) that could help inactivate the invading pathogens. The protective efficacy of the proPO system is further highlighted by the demonstration that gene silencing of PO activating enzymes in *Penaeus monodon* increases the susceptibility of the host to *V. harveyi* infection (Amparyup et al., 2009; Charoensapsri et al., 2009). Given these facts and the demonstrated phenotypes of *V. campbellii* Δ*hmgA*, we suggest that pyomelanization may reduce this bacterium's virulence potential in two ways. First, pyomelanin production by Δ*hmgA* cells may effectively add another layer to the host-assembled melanin around the sites of infection so as to further limit bacterial growth. The comparatively poor survival of Δ*hmgA* in late stationary phase (the time of melanization) (Figure 2A) may allude to this possibility. Second, the pyomelanin produced by Δ*hmgA* may be recognized by the host as another foreign moiety that could further stimulate the proPO system and enhance the clearance of these bacteria.

### Transcriptome analyses

**Figure 3:**
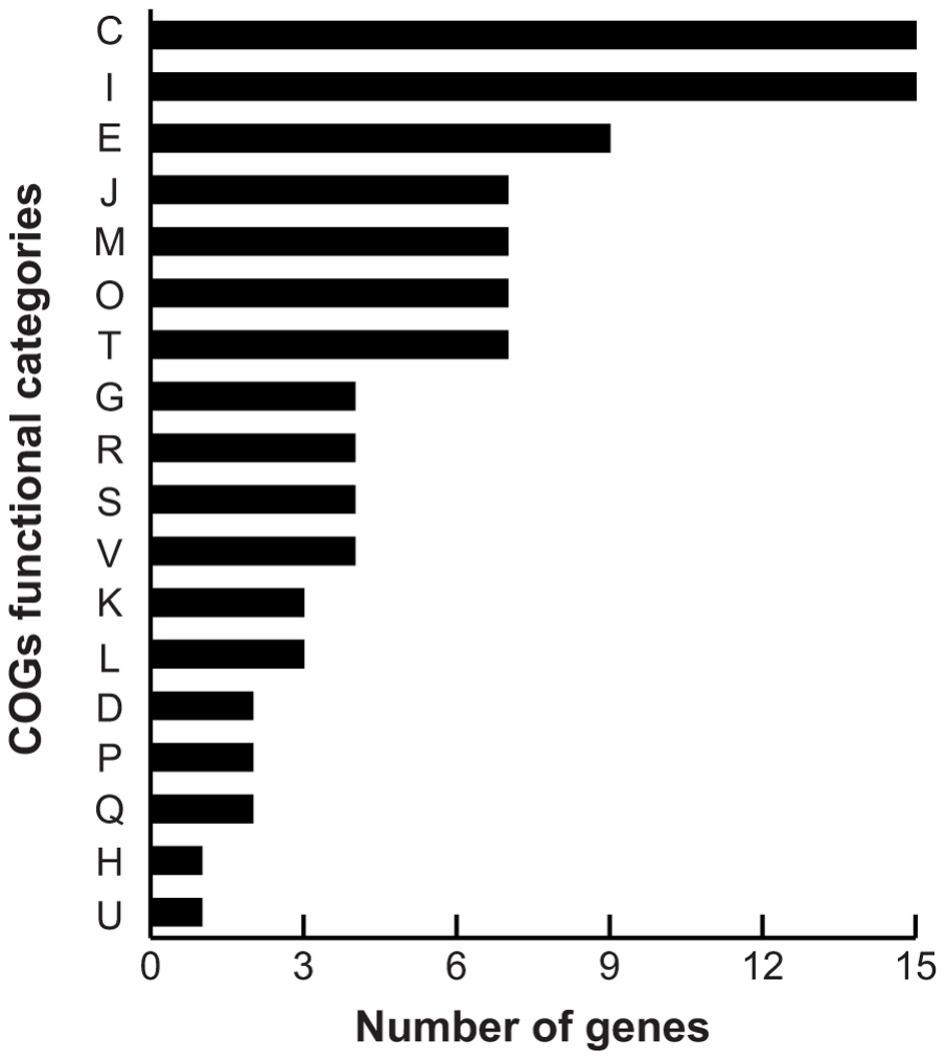
**COG summary of differentially expressed genes**. COG functional category: C, energy production and conversion; I, lipid transport and metabolism; E, amino acid transport and metabolism; J, translation, ribosomal structure and biogenesis; M, cell wall/membrane/envelope biogenesis; O, post-translational modification, protein turnover, chaperones; T, signal transduction mechanisms; G, carbohydrate transport and metabolism; R, general function prediction only; S, function unknown; V, defense mechanisms; K, transcription; L, replication, recombination and repair; D, cell cycle control, cell division, chromosome partitioning; P, inorganic ion transport and metabolism; Q, secondary metabolites biosynthesis, transport and catabolism; H, coenzyme transport and metabolism; U, intracellular trafficking, secretion, and vesicular transport.

As the differences in pyomelanin production, bioluminescence and survival were observed during late stationary phase (48 h), we chose this time point to perform comparative microarray-based expression profiling analyses to understand how the deletion of the *hmgA* gene and subsequent production of pyomelanin gave rise to the observed phenotypes. Whole genome expression profiling revealed that 129 genes (2.7% of the interrogated genome) were significantly modulated in Δ*hmgA* when compared to the WT (adjusted *p*-value < 0.001). Overall, inactivation of the *hmgA* gene appeared to affect the expression of genes involved in energy production and conversion, amino acid metabolism, lipid metabolism, and quorum sensing/bioluminescence (Figure 3). Interestingly, the transcript levels of all 129 genes were found to be less abundant in Δ*hmgA* with approximately 70% of them demonstrating a ≥ twofold reduction in transcript abundance (Table 2). The transcriptional modulation of 10 of these genes was also verified using quantitative RT-PCR (Table 2).

**Table 2:** **Differentially expressed genes in Δ*hmgA* compared with WT *V. campbellii***.

**Gene ID**	**Annotation**	***p*val**	**WT^a^**	**Δ*hmgA***	**Change^b^**	**qRT-PCR^c^**	**Function^e^**
M892_05385	Pyruvate-formate lyase	3.12E-06	2.45	1.11	2.21		C
M892_06250	2-oxoglutarate dehydrogenase	5.20E-04	1.29	0.67	1.93		C
M892_06265	Succinate dehydrogenase	1.59E-04	0.82	0.37	2.22		C
M892_06275	Citrate synthase	1.57E-06	2.31	0.72	3.21		C
M892_08460	Ubiquinol-cytochrome c reductase	1.12E-05	1.45	0.61	2.38		C
M892_08470	Ubiquinol-cytochrome c reductase, iron-sulfur subunit	4.80E-06	1.36	0.86	1.58	2.50	C
M892_10625	ATP synthase subunit alpha	1.24E-04	2.17	1.55	1.40		C
M892_13885	Aconitate hydratase	2.42E-06	2.21	1.12	1.97		C
M892_17240	Cytochrome c-type protein NrfB	1.17E-06	1.24	0.04	31.00		C
M892_17245	Nitrite reductase, Fe-S protein NrfC	1.73E-06	1.24	0.02	62.00	1.58	C
M892_23815	acyl-CoA dehydrogenase, short-chain specific EtfA	6.43E-06	1.72	0.52	3.31	5.30	C
M892_23820	Electron transfer flavoprotein subunit beta EtfB	5.49E-05	1.69	0.73	2.32		C
M892_23825	Electron transfer flavoprotein-ubiquinone oxidoreductase EtfD	5.04E-04	0.85	0.24	3.54		C
M892_24165	Trimethylamine-N-oxide reductase 1	3.42E-06	2.20	1.19	1.85		C
M892_08360	Cell division GTPase	2.15E-05	1.42	0.9	1.58		D
M892_08365	Cell division protein FtsA	3.06E-04	1.13	0.79	1.43		D
M892_03740	Aspartate aminotransferase	2.04E-04	0.90	0.32	2.81		E
M892_03990	Phosphoribosyl-AMP cyclohydrolase	2.70E-05	1.57	0.87	1.80		E
M892_03995	Imidazoleglycerol-phosphate synthase	4.98E-04	1.51	1.04	1.45		E
M892_12425	Argininosuccinate lyase	1.13E-06	1.94	1.00	1.94		E
M892_13555	Aspartokinase	2.04E-04	0.92	0.07	13.14		E
M892_16070	Lactoylglutathione lyase	5.98E-08	3.09	2.02	1.53		E
M892_27505	Sodium/proline symporter PutP	1.13E-06	2.02	0.78	2.59		E
M892_27510	Delta 1-pyrroline-5-carboxylate dehydrogenase PutC	5.98E-08	3.20	1.87	1.71	4.18	E
M892_27515	Proline dehydrogenase PutB	2.10E-07	3.04	1.27	2.39		E
M892_10225	2-keto-3-deoxy-6-phosphogluconate aldolase	9.40E-04	1.12	0.42	2.67		G
M892_13305	Fructose-bisphosphate aldolase	8.05E-04	2.17	1.05	2.07	1.23	G
M892_19270	Enolase	8.81E-04	0.73	0.06	12.17		G
M892_20700	Phosphomannomutase	2.06E-04	1.86	0.69	2.70		G
M892_09970	Delta-aminolevulinic acid dehydratase	1.28E-04	0.60	0.00	UD^d^		H
M892_15785	Glyceraldehyde 3-phosphate dehydrogenase	5.76E-05	2.46	1.37	1.80	6.60	I
M892_24035	3-hydroxyacyl-CoA dehydrogenase IvdG	1.22E-04	0.78	0.25	3.12		I
M892_24040	3-hydroxyisobutyrate dehydrogenase IvdF	5.61E-06	1.73	0.70	2.47		I
M892_24045	Enoyl-CoA hydratase/isomerase IvdE	9.29E-06	2.06	0.66	3.12		I
M892_24050	3-hydroxyisobutyryl-CoA hydrolase IvdD	2.15E-05	1.73	0.36	4.81		I
M892_24055	Branched-chain acyl-CoA dehydrogenase IvdC	1.59E-04	1.12	0.46	2.43		I
M892_24060	Methylmalonate-semialdehyde dehydrogenase IvdB	1.49E-06	2.18	0.55	3.96	2.49	I
M892_24065	3-ketoacyl-CoA thiolase IvdA	1.98E-05	1.94	0.79	2.46		I
M892_24075	Isovaleryl-CoA dehydrogenase LiuA	3.91E-06	2.13	0.87	2.45		I
M892_24080	Methylcrotonyl-CoA carboxylase carboxyl transferase LiuB	5.84E-05	1.46	0.66	2.21		I
M892_24525	Polyhydroxyalkanoic acid synthase PhaC	1.27E-05	1.05	0.10	10.50		I
M892_24530	PHA granule-associated protein PhaD	5.48E-05	3.07	0.57	5.39		I
M892_24535	Acetyl-CoA acetyltransferase PhaA	4.29E-06	3.25	0.95	3.42	6.61	I
M892_24540	Acetoacetyl-CoA reductase PhaB	9.29E-06	2.97	0.83	3.58		I
M892_16425	Acyl carrier protein	8.93E-06	4.07	1.59	2.56		I
M892_02880	Ribosomal protein L35	5.66E-04	2.01	1.40	1.44		J
M892_02885	Bacterial protein translation initiation factor 3	1.83E-05	2.95	2.39	1.23		J
M892_07900	Ribosome-associated protein Y	6.25E-05	4.21	2.97	1.42		J
M892_12355	Translation elongation factor	8.45E-04	1.71	0.60	2.85		J
M892_12355	Translation elongation factor	4.38E-07	3.54	2.41	1.47		J
M892_12355	Translation elongation factor	1.01E-05	3.41	2.02	1.69		J
M892_14350	Translation elongation factor	1.34E-05	1.90	0.81	2.35		J
M892_08765	Transcriptional regulator	5.57E-07	3.21	1.93	1.66		K
M892_12185	Ribonuclease R	1.24E-05	1.87	0.86	2.17		K
M892_13795	Quorum sensing regulator LuxR	2.78E-05	1.98	0.62	3.19	3.57	K
M892_02740	Bacterial nucleoid DNA-binding protein	1.59E-09	2.83	1.07	2.64		L
M892_11585	DNA-binding protein	2.08E-07	1.37	0.13	10.54		L
M892_12540	Replicative DNA helicase	7.48E-05	1.11	0.63	1.76		L
M892_06105	Membrane-bound lytic murein transglycosylase B	2.70E-05	1.36	0.85	1.60		M
M892_08355	UDP-3-O-3-hydroxymyristoyl N-acetylglucosamine deacetylase	5.65E-04	2.28	1.56	1.46		M
M892_08935	UDP-N-acetylmuramate-L-alanyl-gamma-D-glutamyl-m eso-diaminopimelate ligase	1.23E-04	1.05	0.48	2.19		M
M892_12935	UDP-N-acetylglucosamine 1-carboxyvinyltransferase	4.02E-04	1.06	0.60	1.77		M
M892_14045	Outer membrane protein OmpU	7.81E-05	1.24	0.41	3.02		M
M892_20750	Outer membrane protein OmpA	4.16E-05	3.20	1.66	1.93		M
M892_25630	Outer membrane lipoprotein	1.39E-10	4.43	1.51	2.93		M
M892_00180	Heat shock protein HslJ	5.20E-04	1.95	0.58	3.36		O
M892_00340	Glutaredoxin	4.76E-04	1.29	0.61	2.11		O
M892_07710	Antioxidant, AhpC/Tsa family	2.08E-07	2.20	0.28	7.86		O
M892_07875	ClpB protein	8.45E-04	1.25	0.37	3.38		O
M892_09255	Peptidase	5.78E-04	1.79	0.39	4.59		O
M892_11900	Chaperonin GroEL	1.70E-06	1.14	0.16	7.13		O
M892_12155	Serine protease	1.59E-04	1.45	0.58	2.50		O
M892_05540	Cation transport ATPase	4.53E-06	1.34	0.63	2.13		P
M892_15095	Arsenate reductase	5.66E-04	1.36	0.79	1.72		P
M892_12915	Putative ABC superfamily transport protein	5.26E-05	0.41	0.02	20.50		Q
M892_12920	ABC-type transport system, auxiliary component	1.17E-06	1.86	0.49	3.80		Q
M892_01320	Immunogenic protein	1.77E-05	1.63	0.41	3.98		R
M892_12140	RNA-binding protein Hfq	1.46E-06	2.94	1.96	1.50		R
M892_18180	Ecotin precursor	6.37E-05	1.51	0.99	1.53		R
M892_26640	Hemolysin	6.13E-08	1.93	0.54	3.57		R
M892_05800	Lipoprotein-related protein	6.13E-08	1.20	0.24	5.00		S
M892_08695	Hypothetical protein	3.42E-05	1.09	0.40	2.73		S
M892_13325	Hypothetical protein	4.02E-07	3.90	1.96	1.99		S
M892_24630	Hypothetical protein	7.09E-04	0.78	0.23	3.39		S
M892_05410	Putative serine protein kinase	5.29E-06	2.33	0.44	5.30		T
M892_10150	Universal stress protein	1.95E-04	2.37	1.25	1.90		T
M892_12235	Cyclic AMP receptor protein	1.76E-04	1.24	0.59	2.10		T
M892_13400	Negative regulator of sigma E activity	3.29E-05	2.29	1.48	1.55		T
M892_13405	Periplasmic negative regulator of sigma E	1.17E-05	1.51	0.88	1.72		T
M892_13560	Carbon storage regulator	1.18E-04	1.75	1.05	1.67		T
M892_20245	Universal stress protein A	1.03E-09	3.31	1.71	1.94		T
M892_05075	Translocation protein TolB	3.03E-05	1.18	0.65	1.82		U
M892_05460	ABC transporter, ATP-binding protein	6.12E-04	1.16	0.53	2.19		V
M892_07350	Type I site-specific deoxyribonuclease specificity subunit	7.70E-04	1.94	1.20	1.62		V
M892_07355	Type I restriction-modification system, methyltransferase subunit	1.57E-06	2.19	1.34	1.63		V
M892_20315	Beta-lactamase	1.56E-05	1.48	0.49	3.02		V
M892_03945	Hypothetical protein	5.33E-05	0.36	0.00	UD		
M892_05165	Gonadoliberin III-related protein	9.73E-05	0.78	0.1	7.80		
M892_05170	Hypothetical protein	2.71E-07	1.11	0.32	3.47		
M892_05480	Hypothetical protein	1.76E-04	0.98	0.30	3.27		
M892_07360	Phage transcriptional regulator AlpA	2.58E-04	2.02	0.83	2.43		
M892_10485	NADH dehydrogenase subunit II-related protein	1.66E-08	3.75	2.87	1.31		
M892_12930	BolA/YrbA family protein	2.75E-04	0.85	0.07	12.14		
M892_13365	Hypothetical protein	1.73E-04	3.25	1.17	2.78		
M892_15830	Hypothetical protein	7.46E-05	1.24	0.30	4.13		
M892_16465	Hypothetical protein	7.48E-05	1.52	0.27	5.63		
M892_17010	Hypothetical protein	2.32E-08	2.16	0.35	6.17		
M892_18970	Hypothetical protein	2.82E-05	1.39	0.25	5.56		
M892_19525	Putative lipoprotein	1.86E-08	4.17	1.09	3.83		
M892_19565	Muconate cycloisomerase I	2.36E-04	1.64	0.41	4.00		
M892_19745	Hypothetical protein	3.75E-04	0.39	0.00	UD		
M892_20825	Fatty acid reductase LuxC	9.07E-06	2.64	1.23	2.15		
M892_20830	Myristoyl-ACP-specific thioesterase LuxD	1.01E-05	2.98	1.28	2.33		
M892_20835	Alkanal monooxygenase alpha chain LuxA	4.02E-07	4.00	1.78	2.25		
M892_20840	Alkanal monooxygenase beta chain LuxB	3.43E-04	4.84	2.08	2.33	4.81	
M892_20845	Long-chain-fatty-acid luciferin-component ligase LuxE	5.14E-05	2.83	0.87	3.25		
M892_20850	NAD(P)H-dependent FMN reductase LuxG	4.58E-06	1.78	0.81	2.20		
M892_22630	Peptidase	1.03E-04	1.74	1.05	1.66		
M892_22965	Positive response regulator for Pho regulon	3.60E-06	2.16	0.82	2.63		
M892_24070	Transcriptional regulator LiuR	0.0003	0.25	0.00	UD		
M892_24215	Putative GTPase	4.14E-05	1.14	0.50	2.28		
M892_24655	Hypothetical protein	3.22E-05	4.11	1.48	2.78		
M892_25115	Transcriptional regulator LuxT	2.58E-04	2.96	2.25	1.32		
M892_25430	Azurin	5.16E-04	1.88	1.20	1.57		
M892_25755	ATP-dependent protease	7.73E-04	0.75	0.26	2.88		
M892_25795	Predicted transcriptional regulator protein	8.83E-07	2.37	0.26	9.12		
M892_26535	Putative lipoprotein	1.86E-08	1.76	0.19	9.26		
M892_26800	Hypothetical protein	7.73E-05	1.29	0.68	1.90		

a *Average signal intensity log_2_ values from three experiments*.

b Transcript ratio between WT and ΔhmgA.

c Transcript ratio between WT and ΔhmgA from qRT-PCR data. qRT-PCR Ct values were derived from the average of duplicates from two independent biological samples.

d Undivisible as no transcript was detected from ΔhmgA.

e COG functional categories: C, energy production and conversion. D, cell cycle control, cell division and chromosome partitioning; E, amino acid transport ad metabolism; G, carbohydrate transport and metabolism; H, coenzyme transport and metabolism; I, lipid transport and metabolism; J, translation, ribosomal structure and biogenesis; K, transcription; L, replication, recombination and repair; M, cell wall/membrane/envelop biogenesis; O, post-translational modification, protein turnover and chaperones; P, inorganic ion transport and metabolism; Q, secondary metabolites biosynthesis, transport and catabolism; R, general function prediction only; S, function unknown; T, signal transduction mechanisms; U, intracellular trafficking, secretion and vesicular transport; V, defense mechanisms.

### Energy production and electron transfer

Two of 3 genes (M892_08460, M892_08470) in an operon encoding subunits of ubiquinol-cytochrome c reductase were down-regulated in Δ*hmgA*. This cytochrome complex catalyzes the oxidoreduction of mobile redox components generating an electrochemical potential (Kurowski and Ludwig, 1987). Two other modulated genes, NrfB (M892_17240) and NrfC (M892_17245), encode cytochrome-type components of the electron transfer chain of respiratory nitrite ammonification in γ-proteobacteria (Simon, 2002). This electron transfer reaction usually occurs during anaerobic growth and the electron donor formate is readily formed from pyruvate by pyruvate formate lyase. Coincidently, the gene encoding pyruvate formate lyase (M892_05385) was also repressed in the mutant. In addition, a number of genes in the glycolysis pathway, such as those encoding fructose-bisphosphate aldolase (M892_13305), glyceraldehyde 3-phosphate dehydrogenase (M892_15785) and enolase (M892_19270), were down-regulated as well, and likely result in the reduced production of pyruvate. Since HmgA is not a DNA regulatory factor, these results indicated that the pyomelanin produced in Δ*hmgA* may decrease redox activity by serving as an electron sink which in turn would interfere with electron transfer and further weaken cellular respiration in late stationary phase.

### Amino acid and lipid metabolism

The transcripts from three well characterized gene clusters involved in amino acid and lipid metabolism (*etfABD*, *ivdABCDEFG*, *liuABCDE*) were also significantly down-regulated in Δ*hmgA*. These operons encode enzymes that take part in the branched-chain amino acid (i.e., isoleucine, leucine, valine) degradation pathway that is used for energy production in many proteobacteria (Kazakov et al., 2009). Proteins encoded by *etfABD* catalyze electron transfer from quinones to flavoproteins not only in the leucine degradation pathway but also in the fatty acid degradation pathway. The master regulator of the branched-chain amino acid degradation pathway, LiuR, is encoded by the *liuR* gene which resides between the *liuABCDE* and *ivdABCDEFG* operons. The *liuR* gene was also significantly down-regulated in Δ*hmgA* (*p*-value = 0.0003) suggesting a regulatory mechanism for the pyomelanin-induced retardation of branched-chain amino acid degradation.

It was interesting to note that another amino acid degradation pathway was also affected by the production of pyomelanin. The proline utilization operon *putBCP*, encoding proline dehydrogenase, δ-1-pyroline-5-carboxylate dehydrogenase and permease, respectively, was also down-regulated in Δ*hmgA*. However, the expression of *pruR*, which is adjacent to and has been reported to regulate the *putBCP* operon in *P. aeruginosa* PAO1 (Nakada et al., 2002), did not change. Interestingly, transcription of the *putBCP* operon has been reported to respond to osmotic stress by producing more of the final product glutamate in *V. vulnificus* (Lee et al., 2003) and coincidently, pyomelanin biosynthesis was also demonstrated to be induced by osmotic stress in *V. cholerae* (Coyne and Al-Harthi, 1992). Thus, it is possible that Δ*hmgA* pyomelanin may function as a solute glutamate to counter osmotic stress during late stationary phase thus, alleviating the physiological signal for the increased expression of *putBCP* and resulting in the decreased transcription observed in this study.

### Virulence, quorum sensing, and bioluminescence

Although the virulence mechanisms that contribute to bioluminescent vibriosis are not completely understood, several biomolecules are known to be contributing factors (Austin and Zhang, 2006). The expression of many of these virulence factors, including the type III secretion system (Henke and Bassler, 2004a), extracellular toxin (Manefield et al., 2000), metalloprotease (Mok et al., 2003), siderophore (Lilley and Bassler, 2000), chitinase (Defoirdt et al., 2010), phospholipase, caseinase, and gelatinase (Natrah et al., 2011) are known to be regulated by the quorum sensing master regulator LuxR. Our findings from the shrimp virulence model demonstrated that Δ*hmgA* was less virulent than the WT and led to the suggestion that this phenotype may have been due to the down-regulation of several virulence factors in Δ*hmgA*. However, with the exception of two [hemolysin (M892_26640) and azurin (M892_25430)], the genes encoding these factors were not differentially expressed suggesting that the decreased virulence potential of Δ*hmgA* was not caused by an overt down-regulation of accepted virulence factors.

The expression of bioluminescence has also been associated with virulence in shrimp (Manefield et al., 2000; Phuoc et al., 2009) and indirect evidence has suggested that bioluminescence and a toxic extracellular protein are co-regulated (Manefield et al., 2000). The molecular mechanisms of *Vibrio* quorum sensing and its resulting bioluminescence have been most extensively studied in *V. campbellii* BAA-1116 and the regulation of *luxCDABEGH* gene cluster responsible for bioluminescence is known to be positively regulated by the quorum sensing master regulator LuxR (Bassler et al., 1993; Henke and Bassler, 2004b; Lenz et al., 2004; Waters and Bassler, 2006; Tu and Bassler, 2007). In this study, the bioluminescence output of Δ*hmgA* was found to be significantly attenuated and this phenotype could be attributed to the pronounced down-regulation of *luxCDABEGH* in Δ*hmgA* (Table 2). Furthermore, the gene encoding the high cell density state quorum sensing master regulator LuxR and another quorum sensing regulator (LuxT) (Lin et al., 2000) were also significantly modulated in Δ*hmgA*. It is estimated that this singular effect on *luxR* transcript levels accounted for 40% of the transcriptional modulation seen in Δ*hmgA* as a comparison with the LuxR regulon (manuscript in preparation) revealed 52 modulated genes in common.

In addition to the *lux* bioluminescence genes, LuxR has also been shown to regulate the synthesis of storage polyhydroxybutyrates in bioluminescent vibrios (Miyamoto et al., 1998). Polyhydroxybutyrates, the most characterized member of the polyhydroxyalkanoates, are storage polyesters that are produced and accumulate in the bacterial cytosol in carbon-rich environments when other nutrients are limited (Reddy et al., 2003). When available carbon has been exhausted, these storage polymers can be catabolized for carbon and energy. In *V. campbellii* Δ*hmgA*, the expression of the polyhydroxybutyrate synthesis gene cluster (*phaCDAB*) was markedly reduced in comparison to the WT. Thus, a blockage of the tyrosine catabolism pathway and production of pyomelanin not only retarded amino acid degradation but also kept *V. campbellii* from accumulating carbon storage polymers when in a carbon-rich environment. Evidence of these nutrient management deficiencies could be seen in the late stationary phase Δ*hmgA* cultures (Figure 2A) where depleted carbon supplies may have contributed to diminished survival.

The transcriptional modulation of members of the LuxR quorum sensing regulon in Δ*hmgA*, presumably due to reduced *luxR* transcript levels, was unexpected as HmgA is not known to be a regulatory protein. One possible explanation for this observation may be that extracellular pyomelanin is somehow interfering with the binding of autoinducer molecules to their cognate quorum sensing histidine kinase receptors. Alternatively, membrane-embedded pyomelanin may sufficiently alter membrane structure and impair the binding of autoinducer molecules or subsequent phosphorylation cascade. In either case, the quorum sensing signaling cascade would mimic a low cell density state resulting in the phosphorylation of the response regulator LuxO and activated transcription of regulatory small RNAs (Qrr sRNAs) (Lilley and Bassler, 2000). As the base-pairing of the Qrr sRNAs to *luxR* transcripts results in the degradation of *luxR* mRNA (Lenz et al., 2004; Tu and Bassler, 2007), pyomelanization may lock the cells in a low cell density state thus, explaining the transcriptome profiling data.

## Conclusions

In this study, we used the model quorum sensing bacterium *V. campbellii* BAA-1116 to determine how the deletion of the *hmgA* gene and resulting pyomelanization affected cellular phenotypes, virulence, and transcription. While the material properties of *V. campbellii* pyomelanin were similar to previous descriptions, there did not appear to be a generality of pyomelanin-mediated phenotypes as pyomelanization had either a neutral or deleterious effect on cell survival. Despite the fact that pyomelanin production in other pathogenic bacteria had been shown to increase their virulence and adaptation to chronicity in mammalian infection models, our results using a shrimp infection model indicated that pyomelanized *V. campbellii* were actually less virulent than the isogenic WT strain. These observations may be due in part to the comparatively lesser amount of pyomelanin produced and retained in *V. campbellii* and the effect this production had on cellular metabolism. This was supported by the first transcriptome-level analysis comparing a pyomelanin-producing mutant with its isogenic WT strain. It is also worth noting that the immune responses from different animal models (invertebrate vs. vertebrate) may also play a large role in the differences seen in virulence. *V. campbellii* is primarily an invertebrate animal pathogen and this is the first time that the effect of pyomelanization has been tested in an invertebrate model system (i.e., a natural host organism). The transcriptional profiles demonstrated that the deletion of the *hmgA* gene led to significantly lower transcript abundance levels of several important metabolic processes that disrupted cellular homeostasis and fitness in stressful environments (e.g., stationary phase). Taken together, these findings may explain why naturally pyomelanized *V. campbellii* or *V. harveyi* have not been identified from the marine environment or infected eukaryotic host organisms.

### Conflict of interest statement

The authors declare that the research was conducted in the absence of any commercial or financial relationships that could be construed as a potential conflict of interest.

## References

[B1] Amparyup P. Charoensapsri W. Tassanakajon A. (2009). Two prophenoloxidases are important for the survival of *Vibrio harveyi* challenged shrimp *Penaeus monodon*. Dev. Comp. Immunol. 33, 247–256 10.1016/j.dci.2008.09.003 18834900

[B2] Austin B. Zhang X. H. (2006). *Vibrio harveyi*: a significant pathogen of marine vertebrates and invertebrates. Lett. Appl. Microbiol. 43, 119–124 10.1111/j.1472-765X.2006.01989.x 16869892

[B3] Bassler B. L. (1999). How bacteria talk to each other: regulation of gene expression by quorum sensing. Curr. Opin. Microbiol. 2, 582–587 10.1016/S1369-5274(99)00025-9 10607620

[B4] Bassler B. L. Wright M. Showalter R. E. Silverman M. R. (1993). Intercellular signalling in *Vibrio harveyi*: sequence and function of genes regulating expression of luminescence. Mol. Microbiol. 9, 773–786 10.1111/j.1365-2958.1993.tb01737.x 8231809

[B5] Cerenius L. Soderhall K. (2004). The prophenoloxidase-activating system in invertebrates. Immunol. Rev. 198, 116–126 10.1111/j.0105-2896.2004.00116.x 15199959

[B6] Charoensapsri W. Amparyup P. Hirono I. Aoki T. Tassanakajon A. (2009). Gene silencing of a prophenoloxidase activating enzyme in the shrimp, *Penaeus monodon*, increases susceptibility to *Vibrio harveyi* infection. Dev. Comp. Immunol. 33, 811–820 10.1016/j.dci.2009.01.006 19428482

[B7] Coyne V. E. Al-Harthi L. (1992). Induction of melanin biosynthesis in *Vibrio cholerae*. Appl. Environ. Microbiol. 58, 2861–2865 1444398 10.1128/aem.58.9.2861-2865.1992 PMC183019

[B8] Czyz A. Plata K. Wegrzyn G. (2002). Induction of light emission by luminescent bacteria treated with UV light and chemical mutagens. J. Appl. Genet. 43, 377–389 12177528

[B9] Czyz A. Wrobel B. Wegrzyn G. (2000). *Vibrio harveyi* bioluminescence plays a role in stimulation of DNA repair. Microbiology 146, 283–288 10708366 10.1099/00221287-146-2-283

[B10] Defoirdt T. Darshanee Ruwandeepika H. A. Karunasagar I. Boon N. Bossier P. (2010). Quorum sensing negatively regulates chitinase in *Vibrio harveyi*. Environ. Microbiol. Rep. 2, 44–49 10.1111/j.1758-2229.2009.00043.x 23765997

[B11] Defoirdt T. Verstraete W. Bossier P. (2008). Luminescence, virulence and quorum sensing signal production by pathogenic *Vibrio campbellii* and *Vibrio harveyi* isolates. J. Appl. Microbiol. 104, 1480–1487 10.1111/j.1365-2672.2007.03672.x 18070032

[B12] Henke J. M. Bassler B. L. (2004a). Quorum sensing regulates type III secretion in *Vibrio harveyi* and *Vibrio parahaemolyticus*. J. Bacteriol. 186, 3794–3805 10.1128/JB.186.12.3794-3805.2004 15175293 PMC419960

[B13] Henke J. M. Bassler B. L. (2004b). Three parallel quorum-sensing systems regulate gene expression in *Vibrio harveyi*. J. Bacteriol. 186, 6902–6914 10.1128/JB.186.20.6902-6914.2004 15466044 PMC522208

[B14] Ji P. F. Yao C. L. Wang Z. Y. (2011). Reactive oxygen system plays an important role in shrimp *Litopenaeus vannamei* defense against *Vibrio parahaemolyticus* and WSSV infection. Dis. Aquat. Organ. 96, 9–20 10.3354/dao02373 21991661

[B15] Katsev A. M. Wegrzyn G. Szpilewska H. (2004). Effects of hydrogen peroxide on light emission by various strains of marine luminescent bacteria. J. Basic Microbiol. 44, 178–184 10.1002/jobm.200310330 15162391

[B16] Kazakov A. E. Rodionov D. A. Alm E. Arkin A. P. Dubchak I. Gelfand M. S. (2009). Comparative genomics of regulation of fatty acid and branched-chain amino acid utilization in proteobacteria. J. Bacteriol. 191, 52–64 10.1128/JB.01175-08 18820024 PMC2612455

[B17] Kotob S. I. Coon S. L. Quintero E. J. Weiner R. M. (1995). Homogentisic acid is the primary precursor of melanin synthesis in *Vibrio cholerae*, a *Hyphomonas* strain, and *Shewanella colwelliana*. Appl. Environ. Microbiol. 61, 1620–1622 7747976 10.1128/aem.61.4.1620-1622.1995 PMC167418

[B18] Kozakiewicz J. Gajewska M. Lyzen R. Czyz A. Wegrzyn G. (2005). Bioluminescence-mediated stimulation of photoreactivation in bacteria. FEMS Microbiol. Lett. 250, 105–110 10.1016/j.femsle.2005.06.047 16040205

[B19] Kurowski B. Ludwig B. (1987). The genes of the Paracoccus denitrificans bc1 complex. Nucleotide sequence and homologies between bacterial and mitochondrial subunits. J. Biol. Chem. 262, 13805–13811 2820981

[B20] Lee J. H. Park N. Y. Lee M. H. Choi S. H. (2003). Characterization of the *Vibrio vulnificus putAP* operon, encoding proline dehydrogenase and proline permease, and its differential expression in response to osmotic stress. J. Bacteriol. 185, 3842–3852 10.1128/JB.185.13.3842-3852.2003 12813078 PMC161561

[B21] Lenz D. H. Mok K. C. Lilley B. N. Kulkarni R. V. Wingreen N. S. Bassler B. L. (2004). The small RNA chaperone Hfq and multiple small RNAs control quorum sensing in *Vibrio harveyi* and *Vibrio cholerae*. Cell 118, 69–82 10.1016/j.cell.2004.06.009 15242645

[B22] Lilley B. N. Bassler B. L. (2000). Regulation of quorum sensing in *Vibrio harveyi* by LuxO and sigma-54. Mol. Microbiol. 36, 940–954 10.1046/j.1365-2958.2000.01913.x 10844680

[B23] Lin B. Wang Z. Malanoski A. P. O'Grady E. A. Wimpee C. F. Vuddhakul V. (2010). Comparative genomic analyses identify the *Vibrio harveyi* genome sequenced strains BAA-1116 and HY01 as *Vibrio campbellii*. Environ. Microbiol. Rep. 2, 81–89 10.1111/j.1758-2229.2009.00100.x 20686623 PMC2912166

[B24] Lin Y. H. Miyamoto C. Meighen E. A. (2000). Cloning and functional studies of a *luxO* regulator LuxT from *Vibrio harveyi*. Biochim. Biophys. Acta 1494, 226–235 10.1016/S0167-4781(00)00236-0 11121579

[B25] Manefield M. Harris L. Rice S. A. De Nys R. Kjelleberg S. (2000). Inhibition of luminescence and virulence in the black tiger prawn (*Penaeus monodon*) pathogen *Vibrio harveyi* by intercellular signal antagonists. Appl. Environ. Microbiol. 66, 2079–2084 10.1128/AEM.66.5.2079-2084.2000 10788385 PMC101458

[B26] Metcalf W. W. Jiang W. Daniels L. L. Kim S. K. Haldimann A. Wanner B. L. (1996). Conditionally replicative and conjugative plasmids carrying *lacZ* alpha for cloning, mutagenesis, and allele replacement in bacteria. Plasmid 35, 1–13 10.1006/plas.1996.0001 8693022

[B27] Miyamoto C. M. Sun W. Meighen E. A. (1998). The LuxR regulator protein controls synthesis of polyhydroxybutyrate in *Vibrio harveyi*. Biochim. Biophys. Acta 1384, 356–364 10.1016/S0167-4838(98)00028-4 9659397

[B28] Mok K. C. Wingreen N. S. Bassler B. L. (2003). *Vibrio harveyi* quorum sensing: a coincidence detector for two autoinducers controls gene expression. EMBO J. 22, 870–881 10.1093/emboj/cdg085 12574123 PMC145445

[B29] Nakada Y. Nishijyo T. Itoh Y. (2002). Divergent structure and regulatory mechanism of proline catabolic systems: characterization of the *putAP* proline catabolic operon of *Pseudomonas aeruginosa* PAO1 and its regulation by PruR, an AraC/XylS family protein. J. Bacteriol. 184, 5633–5640 10.1128/JB.184.20.5633-5640.2002 12270821 PMC139622

[B30] Natrah F. M. Ruwandeepika H. A. Pawar S. Karunasagar I. Sorgeloos P. Bossier P. (2011). Regulation of virulence factors by quorum sensing in *Vibrio harveyi*. Vet. Microbiol. 154, 124–129 10.1016/j.vetmic.2011.06.024 21775075

[B31] Phuoc L. H. Defoirdt T. Sorgeloos P. Bossier P. (2009). Virulence of luminescent and non-luminescent isogenic vibrios toward gnotobiotic *Artemia franciscana* larvae and specific pathogen-free *Litopenaeus vannamei* shrimp. J. Appl. Microbiol. 106, 1388–1396 10.1111/j.1365-2672.2008.04107.x 19187135

[B32] Reddy C. S. Ghai R. Rashmi Kalia V. C. (2003). Polyhydroxyalkanoates: an overview. Bioresour. Technol. 87, 137–146 10.1016/S0960-8524(02)00212-2 12765352

[B33] Reed L. J. Muench H. (1938). A simple method of estimating fifty percent end point. Am. J. Hyg. 27, 493–497 12177528

[B34] Rodriguez-Rojas A. Mena A. Martin S. Borrell N. Oliver A. Blazquez J. (2009). Inactivation of the *hmgA* gene of *Pseudomonas aeruginosa* leads to pyomelanin hyperproduction, stress resistance and increased persistence in chronic lung infection. Microbiology 155, 1050–1057 10.1099/mic.0.024745-0 19332807

[B35] Rubin R. A. (2009). A first principles approach to differential expression in microarray data analysis. BMC Bioinform. 10:292 10.1186/1471-2105-10-292 19758448 PMC2749840

[B36] Schmaler-Ripcke J. Sugareva V. Gebhardt P. Winkler R. Kniemeyer O. Heinekamp T. (2009). Production of pyomelanin, a second type of melanin, via the tyrosine degradation pathway in *Aspergillus fumigatus*. Appl. Environ. Microbiol. 75, 493–503 10.1128/AEM.02077-08 19028908 PMC2620705

[B37] Simon J. (2002). Enzymology and bioenergetics of respiratory nitrite ammonification. FEMS Microbiol. Rev. 26, 285–309 10.1111/j.1574-6976.2002.tb00616.x 12165429

[B38] Szpilewska H. Czyz A. Wegrzyn G. (2003). Experimental evidence for the physiological role of bacterial luciferase in the protection of cells against oxidative stress. Curr. Microbiol. 47, 379–382 10.1007/s00284-002-4024-y 14669913

[B39] Thompson F. L. Iida T. Swings J. (2004). Biodiversity of vibrios. Microbiol. Mol. Biol. Rev. 68, 403–431 10.1128/MMBR.68.3.403-431.2004 15353563 PMC515257

[B40] Tu K. C. Bassler B. L. (2007). Multiple small RNAs act additively to integrate sensory information and control quorum sensing in *Vibrio harveyi*. Genes Dev. 21, 221–233 10.1101/gad.1502407 17234887 PMC1770904

[B41] Turick C. E. Beliaev A. S. Zakrajsek B. A. Reardon C. L. Lowy D. A. Poppy T. E. (2009). The role of 4-hydroxyphenylpyruvate dioxygenase in enhancement of solid-phase electron transfer by *Shewanella oneidensis* MR-1. FEMS Microbiol. Ecol. 68, 223–225 10.1111/j.1574-6941.2009.00670.x 19573203

[B42] Turick C. E. Tisa L. S. Caccavo F. Jr. (2002). Melanin production and use as a soluble electron shuttle for Fe(III) oxide reduction and as a terminal electron acceptor by *Shewanella algae* BrY. Appl. Environ. Microbiol. 68, 2436–2444 10.1128/AEM.68.5.2436-2444.2002 11976119 PMC127558

[B43] Valeru S. P. Rompikuntal P. K. Ishikawa T. Vaitkevicius K. Sjoling A. Dolganov N. (2009). Role of melanin pigment in expression of *Vibrio cholerae* virulence factors. Infect. Immun. 77, 935–942 10.1128/IAI.00929-08 19103773 PMC2643646

[B44] Wang R. Wang H. Zhou H. Wang Y. Yue J. Diao B. (2011). Characters of homogentisate oxygenase gene mutation and high clonality of the natural pigment-producing *Vibrio cholerae* strains. BMC Microbiol. 11:109 10.1186/1471-2180-11-109 21592381 PMC3114702

[B45] Warrens A. N. Jones M. D. Lechler R. I. (1997). Splicing by overlap extension by PCR using asymmetric amplification: an improved technique for the generation of hybrid proteins of immunological interest. Gene 186, 29–35 10.1016/S0378-1119(96)00674-9 9047341

[B46] Waters C. M. Bassler B. L. (2006). The *Vibrio harveyi* quorum-sensing system uses shared regulatory components to discriminate between multiple autoinducers. Genes Dev 20, 2754–2767 10.1101/gad.1466506 17015436 PMC1578700

[B47] Youngchim S. Morris-Jones R. Hay R. J. Hamilton A. J. (2004). Production of melanin by *Aspergillus fumigatus*. J. Med. Microbiol. 53, 175–181 10.1099/jmm.0.05421-0 14970241

[B48] Zughaier S. M. Ryley H. C. Jackson S. K. (1999). A melanin pigment purified from an epidemic strain of *Burkholderia cepacia* attenuates monocyte respiratory burst activity by scavenging superoxide anion. Infect. Immun. 67, 908–913 9916107 10.1128/iai.67.2.908-913.1999 PMC96403

